# Awareness of long-term foot complications in people living with diabetes mellitus in Mauritius: a cross-sectional study

**DOI:** 10.11604/pamj.2025.51.40.47476

**Published:** 2025-06-10

**Authors:** Sameenah Khodabux, Indrajit Banerjee

**Affiliations:** 1 Department of Cardiology, Dr. Abdool Gaffoor Jeetoo Hospital, Port Louis, Mauritius,; 2 Department of Pharmacology, Sir Seewoosagur Ramgoolam Medical College, Belle Rive, Mauritius

**Keywords:** Diabetes mellitus, endocrine system diseases, Indian Ocean islands, awareness, diabetic foot

## Abstract

**Introduction:**

Mauritius has a high disease burden of diabetes mellitus and ranked twelfth globally according to the International Diabetes Federation. According to the non-communicable disease survey 2021, it was found that 19.9 % of the Mauritian population suffers from Type 2 diabetes mellitus. The long-term diabetic foot complications contribute to premature mortality and increased morbidity. Proper knowledge of diabetic foot complications and good foot care practices may help mitigate the long-term burden of diabetic foot complications in Mauritius in particular and Africa in general. The main objective of this study was to evaluate the awareness of long-term diabetic foot complications and practice of proper foot care among people living with DM in Mauritius.

**Methods:**

a cross-sectional study was conducted among people living with Diabetes Mellitus (DM) attending Non-Communicable Disease (NCD) clinics at Dr. Abdool Gaffoor Jeetoo Hospital (regional hospital), Mauritius and its nearby primary healthcare centers, which serve a majority of the urban and semi-urban population.

**Results:**

the study showed good knowledge about diabetic foot complications and average foot care practicing among the participants. A significant positive correlation between knowledge and practice scores (r = 0.477, p < 0.05) was found, indicating that increased knowledge was associated with better foot care practices. There was a significant negative association between practice score and glycaemic control (r= -0.311 p< 0.001) as well as a negative association between knowledge score and HbA1c. (r=-0.226, p<0.001). It can be deduced that people living with DM with controlled HbA1c had better knowledge about diabetic foot complications and practiced good foot care. The work status of a patient with DM had a significantly better coefficient on knowledge score OR 7.534 [1.392-40.779]. This was found to be statistically significant (p < 0.05). This implies that as the work status of people living with diabetes mellitus improves, the person is 7.534 times more likely to have improved knowledge of diabetic foot complications.

**Conclusion:**

despite having good knowledge on long-term diabetic foot complications, the participants did not practice proper foot care, which could be a result of a lack of education on foot care among people living with DM or a lack of motivation from healthcare professionals, relatives and friends.

## Introduction

Diabetes mellitus (DM) is a long-lasting metabolic disease characterized by persistent hyperglycemia caused by a defect in either insulin secretion by the pancreas or uptake of insulin, or both. It is broadly classified as type 1 and type 2 diabetes mellitus, with the latter accounting for 90% of cases [1-3]. According to the World Health Organization (2022), currently 830 million people around the world are suffering from DM. About two million deaths have been reported globally due to DM and its complications, such as diabetes-related kidney diseases [4]. Mauritius is a small island in the Indian Ocean with a population of 1.2 million people. It has a high burden of non-communicable diseases such as diabetes mellitus and ranked twelfth globally according to the International Diabetes Federation. As per the World Health Organization´s (WHO) biennium report 2022-2023, the diagnosis and management of DM is a real public health issue in Mauritius [5]. The non-communicable disease (NCD) survey 2021 conducted on the island showed that 19.9 % of the Mauritian population suffers from Type 2 Diabetes Mellitus (T2DM). The prevalence in men and women is 21.6% and 18.5%, respectively. Thirty-one point seven percent (31.7%) of people living with diabetes had HbA1c ≥ 9.0, which indicated poor glycaemic control and a major part of the population was at an increased risk of developing diabetic complications [5]. Diabetes is the second most common leading cause of death (20%) among people after ischaemic heart disease (21.3%) in Mauritius (2021) [6,7].

A study by Edmonds *et al*. on diabetic foot ulcer (DFU) reported a probability of 19% -34% people living with DM developed DFU over a lifetime. Diabetic foot ulcer is likely to recur after initial healing and it has been shown that its occurrence increases yearly. Osteomyelitis develops in about 20% of DFU and complicated diabetic foot infections end in lower limb amputations [8]. About 131 million people suffered from lower limb complications due to DM in 2016. 2.1% of the years lived with disability, representing an estimated 16.8 million people. Assessments show that these complications are a huge and growing predisposing factor to the disability problem across the world and affect the middle to older aged male populations [9]. Diabetic foot complications are more common in developing countries due to poorly controlled DM and a lack of foot care and education. The American Diabetes Association in 2020 suggested that people living with DM should have a far-reaching foot assessment once per year, and there is an absolute need to set up preventive measures to decrease the financial and psychological burden on the people and the country [10]. The need for this research arises from the circumstances that a dearth of information and data related to knowledge on long-term diabetic foot complications and good foot care practices in people with DM prevails in Mauritius.

The focus of this study is to decrease the number of people with poorly controlled diabetes and hence prevent or delay the complications related to DM and improve the quality of life. Moreover, these complications usually have a detrimental effect on the physical and psychological aspects of the health of the patients as well as cause a financial burden on the economy of the country [7]. The primary objective of this study was to evaluate the knowledge and attitudes on long-term diabetic foot complications such as non-healing wounds, diabetic foot ulcers, amputations and proper foot care practices among people with diabetes mellitus in Mauritius. Furthermore, the findings of this study will help in the prevention of these complications by providing information to healthcare workers, the Ministry of Health and Wellness, policymakers and other non-governmental organizations. The burden of diabetes related foot disease (DFD) is believed to rise in the future. People´s education and high-risk factor determination, appropriate assessment, and new pharmacological strategies will decrease the risk of DFD and the disease burden in Mauritius. To the best of our knowledge, this will be the first study determining knowledge and practice on long-term diabetic foot complications of diabetes mellitus among people living with DM, conducted and reported in Mauritius.

## Methods

**Study design:** a cross-sectional study was conducted to assess the awareness of long-term foot complications in people aged 18 years and above, living with diabetes mellitus in Mauritius. A quantitative study was designed and reported according to Strengthening the Reporting of Observational Studies in Epidemiology (STROBE) guidelines.

**Study setting:** the study was carried out in the non-communicable disease (NCD) clinics of Dr. A. G. Jeetoo Hospital in Port Louis, the capital of Mauritius, and its surrounding primary healthcare centres, which serve a majority of the urban and semi-urban population. It is one of the major regional hospitals out of five in Mauritius situated in the district of Port Louis. This hospital has a wider population coverage, and it is expected that most of the patients living with DM in the catchment area and the periphery of the capital will report to the hospital.

**Data collection:** data collection was done between 20^th^ June 2021 and 15^th^ September 2021 by the primary investigator (SK) in person during NCD clinics. Data were gathered through a structured interview questionnaire of closed-ended questions, which lasted for about 10 minutes, and only HbA1c and year of diagnosis of DM were obtained from patient´s medical file.

**Inclusion criteria:** people aged 18 years and above, living with diabetes mellitus, and consenting to participate were included in the study. The participants were people living with DM attending their regular NCD follow-up either at Dr. Abdool Gaffoor Jeetoo Hospital or nearby healthcare centres. A probability sampling method was used whereby every third patient with diabetes mellitus meeting the inclusion criteria was selected to participate in this study. A systematic sampling method was used for data collection. Sampling interval (k) was determined by: k=N/n; N: Population size 1200, n: desired sample size (1200/384=3.125). Thus, every 3^rd^ person living with DM was selected and included in the study. Randomization was selected at the starting point between 1 and 3.

**Exclusion criteria:** people with gestational diabetes and with traumatic ulcers resulting from other causes, such as road traffic accidents, were excluded from this study, as well as people refusing to give consent for participation.

**Outcome variable:** the main outcome variables were knowledge of diabetic foot complications and practice of foot care among people living with DM in Mauritius. For knowledge (uncontrolled DM, noncompliance to medications, patients' diet, exercise, foot ulcers and fungal infections, smoking and diabetes, lack of sensation in their feet) and practice of foot care (self-examination of feet, washing of feet, drying between toes, using moisturizing cream or lotion on feet, moisturizing cream or lotion between toes, toenails cut straight, changing of socks, checking of shoes, checking feet are dry after washing, checking the temperature of the water to wash feet, dry dressing on a blister/wound, report to the doctor when have an injury, walk around the house in bare feet, walk outside the house in bare feet, wearing shoes without socks) were used.

**Explanatory variable:** factors that were taken into account on the individual level were gender (male, female), age (18-35 years, 36-45 years, 46-65 years, above 65 years), education level (illiterate, primary, secondary and tertiary), working status (employed, unemployed, retired, household duties), marital status (married, single, divorced, widowed), smoker (daily, sometimes, rarely, never), alcohol intake (daily, sometimes, rarely, and never). Type of diabetes mellitus (type 1 and type 2), duration of diabetes mellitus (< 5 years, 5-10 years, more than 10 years), treatment (oral antidiabetic drug, insulin, combination of oral antidiabetic drug and insulin, and lifestyle modifications).

**Scoring scale:** the categorical data recorded on a Likert scale and dichotomous scales were coded. There were 8 items to determine the knowledge of diabetic foot complications. Closed-ended questions were asked to the participants to assess their knowledge on diabetic foot complications and the answers were scored on a 4 point- Likert scale as Yes (3), Maybe (2), Don´t know (1) and No (0). Total score for assessment of knowledge about diabetic foot complications was 24. A total score of 12 or above was considered to be “Yes” and below 12 it was considered to be “No" for knowledge domain. For the practice domain, 16 items were used. The answers were recorded on a 4-point Likert scale as well: often (3), sometimes (2), rarely (1) and never (0). A reverse scoring was used for the questions reflecting bad foot care practice. Total score for practice of foot care was 48. A total score of 24 or above was considered to be Yes and below 24 it was considered to be No for the practice domain. By combining both of these scores, the overall score was 72. A high score was defined as a percentage score of 70% and above which meant good knowledge on diabetic foot complications and good foot care practices, a middle score as 40-70% and a poor score below 40%. The questionnaire was partly adapted from pre-validated tools such as the Nottingham assessment of functional foot care questionnaire to assess the practice of foot care, with modifications made to suit the local Mauritian population [11].

**Questionnaire design:** after an extensive review of the literature, a questionnaire was developed so that they were relevant to meet the objectives of the study. The final questionnaire was divided into five sections, namely: A) sociodemographic characteristics, B) diabetes-related information, C) knowledge about diabetic foot care and complications, and D) practice of foot care (partly adapted from Nottingham assessment of functional foot care).

**Questionnaire validity:** four subject matter experts in endocrinology and diabetic care reviewed the tool. Each item was rated for relevance using a 4-point Likert scale, and average congruency was computed as per polit and beck´s method [12]. The questionnaire was modified based on their comments, and an average congruency percentage of 90% was obtained. A pilot study was conducted among 15 people living with DM to determine the face validity and based on their feedback, the questionnaire was modified.

**Reliability analysis:** to assess the level of knowledge of the participants, a knowledge score was computed. A reliability test was carried out which gave Cronbach's alpha of 0.844. For the practice domain, Cronbach's Alpha was found to be 0.629. After removing 2 items from the questionnaire, do you wear half-colored socks? Do you wear artificial material (e.g. Nylon) socks’ the Cronbach's Alpha was found to be 0.712.

**Sample size calculation:** the number of research subjects needed to attain results that could be applied to the general population. It was statistically determined by using the formula:


$$n=\frac{z^{2}p\left(1-p\right)}{d^{2}}$$

n: sample size, z: value of standard normal distribution corresponding to 95% confidence interval, p: expected proportion in the population, d: absolute precision. Where, z=1.96 for 95% Confidence interval, p=0.8, d=0.04.


$$n=\frac{1.96^{2}\times 0.8\left(1-0.8\right)}{0.04^{2}}$$

The sample size was 384 people living with Diabetes Mellitus who were considered for this research. Sample size adequacy and statistical power were assessed before data collection, and a sample size of 384 was determined to be sufficient to detect expected effects with 95% confidence and 5% precision. We have achieved an adequate sample size of 390. To account for potential dropouts or non-responses, we increased the sample size by 10%, resulting in a final target of 390 participants to ensure adequate statistical power.

**Data management and statistical analysis:** the data collected from the questionnaires were inserted on the Statistical Package for the Social Sciences (SPSS) version 28 for data analysis. Before data analysis, the dataset was cleaned by identifying and handling missing values through imputation, removing outliers based on standard deviation thresholds, and resolving inconsistencies through data validation checks. Descriptive analysis was used for participants´ sociodemographic details and diabetes-related information. ANOVA, Multinomial regression analysis, and Pearson Correlation tests were used to compare group means, analyze categorical outcomes with multiple categories, and assess linear relationships between continuous variables, respectively. Before performing the statistical tests, homogeneity of variance for ANOVA and linearity for Pearson correlation were checked. P-value < 0.05 was considered as the level of significance.

**Ethical clearance:** ethical approval was obtained from the Ethics Sub Committee of the Ministry of Health and Wellness on 7^th^ June 2021, reference number: (MHC/CT/NETH/2021). Data were stored securely and anonymously to ensure participant´s confidentiality, in compliance with the Data Protection Act of Mauritius. Informed consent was taken from all the participants, and they were informed that they could withdraw from the study at any point in time and that their information was kept confidential and used for research purposes only.

## Results

**Sociodemographic details of the people living with DM:** among 390 people living with DM, 213 were female (55%) and 177 were male (45%). Most of the people were in the age group 46-65 years: 176 (45%) followed by more than 65 years (26%). Regarding education level and working status, the majority of the participants (182) studied till secondary education (47%) and 210 among them were employed (54%) (Table 1).

**Table 1:** socio-demographic distribution of people 18 years old and above living with diabetes mellitus in Mauritius, June-September 2021 (N = 390 cases)

Sociodemographic details	Frequency	Percentage (%)	Diabetes-related information	Frequency	Percentage (%)
**Gender**			**Type of diabetes mellitus**		
Male	213	55%	Type 1	28	7%
Female	177	45%	Type 2	215	55%
**Age**			Don't Know	147	38%
18-35 years	39	10%	**Duration of diabetes mellitus**		
36-45 years	74	19%	< 5years	120	31%
46-65 years	176	45%	5-10 years	146	37%
Above 65 years	101	26%	More than 10 years	124	32%
**Education level**			**Treatment**		
Illiterate	34	9%	Oral antidiabetic drug	251	64%
Primary	122	31%	Insulin	35	9%
Secondary	182	47%	Both Oral antidiabetic drug and Insulin	74	19%
Tertiary	52	13%	Life>	30	8%
**Working status**			**Do you take your medications regularly?**		
Employed	210	54%	Yes	375	96%
Unemployed	17	4%	No	15	4%
Retired	91	23%	**Do you attend your appointment regularly?**		
Household duties	72	18%	Yes	378	97%
**Marital status**			No	12	3%
Married	257	66%	**Family history of diabetes mellitus**		
Single	47	12%	Yes	335	86%
Divorced	29	7%	No	55	14%
Widowed	57	15%			
**Smoker**			-		
Daily	41	10.5%			
Sometimes	26	6.7%			
Rarely	33	8.5 %			
Never	290	74.4%			
**Alcohol intake**			-		
Daily	1	0.3%			
Sometimes	37	9.5%			
Rarely	123	31.5%			
Never	229	58.7%			

**Diabetes-related information:** in terms of their lifestyle, it is observed that 74.4 % of them do not smoke, while 25.6% are smokers. 58.7 % of the participants do not consume alcohol. People living with DM for 5-10 years: 146 (37%) followed by more than 10 years: 124 (32%). Most of the participants, 251 (64%), were on oral antidiabetic drugs, followed by a combination of both insulin and oral antidiabetic drugs 74 (19%), insulin therapy, 35 (9%), and lifestyle modifications 30 (8%), respectively. Ninety-six percent (96%) participants took their medications regularly, while 97% attended doctors´ appointments regularly. Three hundred thirty-five (335) (86%) people had a positive family history of DM. Among 390 people, 215 were suffering from type 2 diabetes mellitus (55%) (Table 1).

**Knowledge about diabetic foot complications:** the level of knowledge varied from 66% to 95%. 95% of the participants knew that people living with DM should have a balanced diet and exercise. Ninety (90%) knew that uncontrolled DM can cause diabetic foot complications. 83% were aware that DM can cause foot ulcers and fungal infections in the feet. Seventy-five (75%) knew that DM could lead to loss of sensation in the feet. Seventy (70%) knew that foot complications were a consequence of non-compliance whith medications. They were even less aware of the fact that smoking while having DM could lead to blockage of arteries, which could result in poor blood flow to the feet (66%) (Table 2).

**Table 2:** knowledge on diabetic foot complications and practice towards foot care among people of 18 years old and above living with diabetes mellitus in Mauritius, June-September 2021 (N = 390 cases)

Questions	Frequency of answers Yes	Percentages (%)
**Knowledge on diabetic foot complications**		
Uncontrolled diabetes mellitus (DM) can cause diabetic foot complications.	352	90%
Foot complications are the result of not taking medications regularly.	273	70%
Patients with diabetes mellitus should have a balanced diet.	372	95%
People with diabetes mellitus must exercise.	372	95%
Diabetics can develop foot ulcers and fungal infections.	322	83%
Smoking and diabetes cause blockage of the arteries which decreases blood flow to the feet.	256	66%
People living with diabetes mellitus can develop a lack of sensation in their feet.	292	75%
**Practice towards foot care among diabetic patients**		
Do you examine your feet?	297	76%
Do you wash your feet?	390	100%
Do you dry between your toes?	315	81%
Do you use moisturizing cream or lotion on your feet?	227	58%
Do you put moisturizing cream or lotion between your toes?*	88	23%
Are your toenails cut straight?	83	21%
How often do you change your socks?	237	60%
Do you check your shoes before you put them on?	246	63%
Do you check your shoes when you take them off?	137	35%
Do you check your feet are dry after washing?	307	79%
Do you check the temperature of the water you wash your feet in?	115	29%
Do you put a dry dressing on a blister/wound when you get one?	347	89%
Do you report to the doctor when you have an injury to your feet?	374	96%
Do you walk around the house in bare feet?*	192	49%
Do you walk outside the house in bare feet?*	66	17%
Do you wear shoes without socks?*	129	33%

* Note: In bold are bad practices and inverse scoring was used

**Knowledge and attitudes towards the practice of foot care:** seventy-six (76%) of the respondents examined their feet regularly, all the participants (100%) washed their feet, however, only 81% dried between their toes. Fifty-eight (58%) would moisturize their feet using a cream/lotion and only 21% of them would also apply it in between their toes. Checking the temperature of the water before washing feet was not a regular practice among the people (29%). Twenty-one (21%) of the respondents would cut their toenails straight and 60% would change their socks regularly. While 63% of them would check their shoes before putting them on, only 35% would check their shoes after removing them. Eighty-nine (89%) mentioned that they would apply a dry dressing on the injury themselves, while 96% would visit a doctor following an injury in their foot. Thirty-two percent (32%) wore colored socks. Walking barefoot was not a common practice, with 49% doing it inside the house and only 17% outside. Also, most of them (90%) did not wear socks made of synthetic material. One-third of them (33%) wore shoes without socks (Table 2). The knowledge and practice scores, the association between knowledge, practice and glycaemic control (HbA1c) are shown in Table 3. It can be deduced that the knowledge score was high with 336 (86%) of the sample getting a high score, 34 (9%) a middle score and 20 (5%) a poor score. Males achieved more good knowledge scores than females.

**Table 3:** knowledge and practice scores on diabetic foot complications, association between knowledge and practice scores and glycaemic control (HbA1c) among people of 18 years old and above living with diabetes mellitus in Mauritius, June-September 2021 (N = 390 cases)

	Practice scores= n (Percentage%)			Knowledge score = n (Percentage%)		
	High score (70-100%)	Middle score (40-70%)	Low score (below 40%)	High score (70-100%)	Middle score (40-70%)	Low score (below 40%)
**Male**	60 (34%)	111 (63%)	6 (3%)	156 (88%)	16 (9%)	5 (3%)
**Female**	89 (42%)	109 (51%)	15 (7%)	180 (85%)	18 (9%)	15 (7%)
Total	149 (38%)	220 (56%)	21 (5%)	336 (86%)	34 (9%)	20 (5%)
**Association knowledge and practice**		**Practice score**		**Knowledge score**		
Pearson correlation	Practice score	1.000	-	0.477	-	-
	Knowledge score	0.477	-	1.000	-	-
Sig. (1-tailed)	Practice score		-	0.000	-	-
	Knowledge score	0.000	-		-	-
N	Practice score	390	-	390	-	-
	Knowledge score	390	-	390	-	-
		Sum of squares	df	Mean square	F	Sig.
ANOVA results	Regression	16887.881	1	16887.881	114.579	<.001
	Residual	57187.823	388	147.391		
	Total	74075.704	389			
**Association knowledge, practice, and glycaemic control (HbA1c)**		**Last HbA1c**	**Practice score**	**Knowledge score**		
	Pearson correlation	1	-.311**	-.226**		
	Sig. (2-tailed)		0.000	0.000		
	N	390	390	390		
	Pearson correlation	-.311**	1	.477**		
	Sig. (2-tailed)	0.000		0.000		
	N	390	390	390		
	Pearson correlation	-.226**	.477**	1		
	Sig. (2-tailed)	0.000	0.000			
	N	390	390	390		
	Pearson correlation	1	-.311**	-.226**		
	Sig. (2-tailed)		0.000	0.000		
	N	390	390	390		

** Correlation is significant at the 0.01 level (2-tailed)

In the practice score domain, 38% achieved a high score, 51% got a middle score and 5% a poor score. Females achieved higher scores than males in this domain. A significant positive correlation between knowledge and practice scores (r = 0.477, p < 0.05) was found, indicating that increased knowledge was associated with better foot care practices. The ANOVA results show that the regression model is predicting the practice score significantly. There was a significant negative association between practice score and glycaemic control (r= -0.311, p< 0.001) as well as a negative association between knowledge score and HbA1c. (r=-0.226, p<0.001). It can be deduced that people living with DM with controlled HbA1c had better knowledge about diabetic foot complications and practiced good foot care. The mean practice score was 63.2733 ± 13.79949 SD while the mean knowledge score was 86.1538 ± 19.54638 SD. The parameter estimates in Table 4 shows if predictors were significant, that is p-value <0.05 and give the odds ratio of each outcome occurring. A high score was taken as the reference category, since the aim of the study was to assess the factors, which can improve knowledge of DM. The work status of a patient with DM had a significantly better coefficient on knowledge score, which was positive with an odds ratio of 7.534 (95% confidence interval 1.392-40.779). This was found to be statistically significant (p < 0.05).

**Table 4:** classification of knowledge score among people of 18 years old and above living with diabetes mellitus in Mauritius, June-September 2021 (N = 390 cases)

	Classification knowledge scores	B	Std. Error	Wald	df	Sig.	Exp(B)	95% Confidence interval for Exp(B)	
								Lower bound	Upper bound
**Low Score (<40%)**	Age group	0.021	0.599	0.001	1	0.972	0.980	0.303	3.171
	Gender	0.354	0.571	0.386	1	0.535	1.425	0.466	4.362
	Marital status	0.860	0.512	2.820	1	0.093	2.363	0.866	6.444
	Duration of having diabetes mellitus	0.717	0.822	0.761	1	0.383	2.048	0.409	10.254
	Level of education	0.913	0.588	2.411	1	0.120	0.401	0.127	1.270
	Work status	2.019	0.862	5.493	1	0.019	7.534	1.392	40.779
**Middle Score (40-70%)**	Age group	0.046	0.515	0.008	1	0.929	0.955	0.348	2.620
	Gender	0.277	0.386	0.516	1	0.472	0.758	0.356	1.614
	Marital status	0.106	0.390	0.074	1	0.785	1.112	0.518	2.388
	Duration of having diabetes mellitus	0.782	0.528	2.190	1	0.139	2.186	0.776	6.157
	Level of education	0.344	0.599	0.331	1	0.565	0.709	0.219	2.291
	Work status	0.760	0.505	2.266	1	0.132	2.138	0.795	5.750

The reference category is: high score (70-100%)

## Discussion

**Sociodemographic details of the participants:** among 390 people living with DM, the majority (45%) of the participants were aged 46 years and above, and 26% were above 65 years old, and most of the people (n=213) were female (55%). According to the NCD survey 2021, the prevalence of DM (age and sex standardized to the national population of Mauritius in 2008) in adults aged 25-74 years was 19.9% : 21.6% for men and 18.5% for women [6]. Our findings are similar to a study conducted by Qasim *et al*. which showed that the mean age of the people who suffered from diabetes mellitus was 52.49 years [1,3]. A study conducted in India showed that the majority of the people were female 64.0%, as compared to males (36%) [14]. Most of the people were literate, with 91% of them having attained at least primary education. Our findings are parallel to a study conducted by Al Amri *et al*. on 1000 diabetic people, which displayed that most of the people were educated and the majority of them, 56.6% attended university and 23.8% until secondary level of education [15]. It is important to note that in this study, the education level did not have any association with the knowledge score of diabetic foot complications in people living with DM. Many studies showed that knowledge of diabetic foot complications and good foot care practices were associated with education level [15,16]. Only 54% were working members of the active population of Mauritius and 4% were unemployed, 23% were retired persons, and 18% were housewives/househusbands. The work status of a patient with DM had a significantly better coefficient on knowledge score, which was positive with an odds ratio of 7.534 (95% confidence interval 1.392-40.779). This was found to be statistically significant (p < 0.05). This implies that as the work status of people living with Diabetes Mellitus improves, the person is 7.534 times more likely to have improved knowledge of diabetic foot complications. This finding was found to be dissimilar as compared to a study conducted by Ramirez-Perdomo *et al*. in Colombia, which showed that the high percentage of T2DM, was found in homemakers (53.9%) whereas it was only 3% among employed people [17]. In Mauritius, the working population is between 18-65 years old, which is the main population for people living with DM. According to the WHO, one in five Mauritians has type 2 DM [18].

**Diabetes-related information of the participants:** most of the people suffering from T2DM accounted for 55%, 7% from T1DM and 38% were not aware of the type of diabetes mellitus they suffered from. Thirty-one percent (31%) of people had Diabetes Mellitus for less than 5 years, 37% for 5-10 years and 32% were diagnosed for more than 10 years. A study by Lamchahab *et al*. showed that the duration of DM did not affect the knowledge of diabetic foot complications among their participants, which was consistent with this study. The chronic nature of DM may have an impact on the knowledge of the complications of the disease because many physicians think that the longer the disease duration, the better the people are informed [19]. Around two-thirds of the participants (64%) were on oral hypoglycaemic drugs (OHD), 9% were on insulin therapy, 19% were on both OHD and insulin, and 9% were advised to change their lifestyle to control Diabetes Mellitus. 96% of the people interviewed mentioned being regular on their medications, while 97% attended regularly their appointments at the NCD clinics. Sixty-six (66%) of the people living with diabetes mellitus interviewed had a positive family history of diabetes mellitus. These findings were parallel to a study conducted by Paramasivam *et al*. which showed similar results [14].

**Knowledge towards diabetic foot complications and practice of foot care:** the participants had a good knowledge score regarding diabetic foot complications, while they had an average score for the practice of proper foot care practices, and this study also showed that there was a positive relationship between knowledge and practice scores. Pearson correlation analysis showed a significant positive correlation between knowledge and practice scores (r = 0.477, p < 0.05), indicating that increased knowledge was associated with better foot care practices. There was a significant negative association between practice score and glycaemic control (r= -0.311, p< 0.001) as well as a negative association between knowledge score and HbA1c (r=-0.226, p<0.001). It can be deduced that patients with controlled HbA1c had better knowledge about diabetic foot complications and practised good foot care. Good knowledge significantly improved the practice of proper foot care in people living with DM. The practice score was 63% on average while the knowledge score was 86%. This could be explained by the fact that although people were aware of the diabetic foot complications, they were not practising proper foot care, or sufficient information was not available on how to practice proper foot care. The findings of this study are similar to the study conducted by Qasim *et al*. [13] and Muhammad-Lutfi *et al*. [20], which showed the people had better knowledge score and moderate practice score. The scores for good foot practices were found to be lower than those of knowledge [20] whereas a study by Pourkazemi *et al*. showed that most of its participants had lower scores of knowledge and practice about foot care; the mean practice score was lower than the knowledge score [21] which is different from our finding and Ramirez-Perdomo *et al*.showed a poor score for both knowledge and practice for diabetic foot prevention [17].

In this study, 34% of the participants did not know that smoking and diabetes mellitus can lead to blockage of the arteries, resulting in poor blood circulation to the lower limbs. The findings of our study were similar to a study conducted by Shamim *et al*. in Saudi Arabia [22]. In Mauritius, the government has set up tobacco cessation clinics all over the island. It is part of the National Action Plan for Tobacco Control. People living with DM are encouraged by the healthcare professionals to attend these clinics which are free of cost. This eventually builds up their knowledge on the hazards of smoking on their health [23]. A study conducted by Almuhanadi *et al*. reported that women had a better practice score than knowledge score while men had good knowledge but average foot care practices [24]. A cross-sectional survey conducted by Hellenberg *et al*. showed that there was a difference in the attitude of self-care in people living with DM having foot ulcers. Females were more active in practicing proper foot care and looking for more information, and trying to adjust to the situation [25]. Similar findings were found in a study by Darshan *et al*. where females were more concerned about proper foot care practices [26]. The WHO in 2022 recommended the broadening of foot care services over the island, and also helped in training nurses to better address diabetic neurovascular complications of the foot. It also helped with the infrastructure and equipment, improving access and investing in skilled personnel, with a view to decreasing the number of amputations in Mauritius, hence improving the quality of life of people living with DM [18].

In this study it was found that 58% of participants, mainly women, applied moisturizing cream/lotion on their feet while only 23% put moisturizing cream/lotion in between their toes. This was not a common practice among men. In a similar study by Hirpha *et al*. it was reported that 63.5% of the people never applied moisturizing cream and 65.4% never applied moisturizing cream between feet and toes [27]. According to Kirsner *et al*. application of daily moisturizers and cleansers can help to improve skin barrier dysfunction and prevent complications in diabetes mellitus. About 46 % of the participants in this study preferred to wear slippers, followed by sandals 31% [28]. The findings were similar to the study conducted by Hellenberg *et al*. [25]. Among the bad foot care practices, 79% of people living with DM did not know that their toenails should be trimmed straight to prevent injury to their toes. A study conducted by Hasnain *et al*. 2014 showed similar findings [29]. It is also worth noting that Mauritius is a small island of 1.2 million inhabitants and there is no significant difference in cultures and socioeconomic factors that can be seen in the patients attending the public healthcare facilities. A qualitative study would be ideal to understand the essence and behavior of the people living with DM along with their thoughts.

**Limitations of the study:** due to COVID-19 pandemic and lockdown in Mauritius, data collection was done over a short period of time. The behaviour of the participants could not be studied. A mixed-methods approach can be conducted in the future to understand the perception and feeling of people living with DM towards long-term diabetic foot complications and to understand the essence of why they do not practice good foot care practices, although they know long-term diabetic foot complications. Some participants had recall difficulties giving rise to recall bias in the study. They did not exactly have the duration of their DM and the same had to be checked in their folders.

## Conclusion

It was concluded from the study that there was a high degree of knowledge and attitude about diabetic foot complications and average foot care practices among the people living with diabetes mellitus in Mauritius. Multicentric mixed-methods research, such as a qualitative study and/or a quantitative study with a higher sample size, is required in the future to find out the knowledge, attitude, and practice of long-term diabetic foot complications and diabetic foot care as well as to understand the beliefs of the people living with DM.

### What is known about this topic

- Mauritius has a high disease burden of diabetes mellitus and ranked twelfth globally according to the International Diabetes Federation; according to the non-communicable disease (NCD) survey 2021, it was found that 19.9 % of the Mauritian population suffers from Type 2 Diabetes Mellitus (T2DM);
- Diabetes is the second most common leading cause of death (20%) among people after heart disease (21.3%) in Mauritius (2021);
- There is a dearth of information and data related to knowledge and practice related to long-term diabetic foot complications in people with diabetes mellitus in Mauritius.

### What this study adds

- This study has shown that people living with DM have good knowledge about long-term diabetic foot complications but still did not practice good foot care; Mauritius being a country with a high non communicable diseases burden, where every fifth patient is suffering from DM, should develop strategies to help the government and healthcare professionals and other policymakers to address this gap between knowledge and practice;
- The burden of diabetes-related foot disease (DFD) is believed to rise in the future; people’s education and high-risk factor determination, appropriate assessment, and new pharmacological strategies will decrease the risk of DFD and the disease burden in Mauritius thereby decreasing the rate of amputations and improving quality of life of people living with DM;
- The findings of this study will definitely help in the prevention of these complications by providing information to healthcare workers, the Ministry of Health and Wellness, policymakers and other non-governmental organizations.
